# Medical Society of Kentucky

**Published:** 1855-11

**Authors:** 


### MEDICAL SOCIETY OF KENTUCKY.

We wish to remind the physicians of Kentucky that the next annual meeting of the *State Medical Society* will be held in Frankfort, on the first Wednesday in February, and to urge a general attendance of the medical men of the State. Kentucky has an able medical faculty. Among her numerous physicians there are very many men of talents and learning who would be ornaments to the profession any where. But they are isolated, and comparatively unknown. Their fine powers and accomplishments are like a candle hid under a bushel. The meetings of the State Medical Society afford opportunities for discovering their worth of which they ought to avail themselves. We assure them they have lost a great deal in not attending former meetings, and if they are true to their own interests, not to speak of the duty they owe to their profession, they will not neglect those which are to come. These re-unions are exceedingly pleasant, and exert the happiest influence upon the social character of our profession, and in addition to all the rest, they re-kindle those fires of professional ambition and zeal which tend so much to advance and sublimate the profession. We trust that every county and town in the State will be represented at the meeting in Frankfort.

